# Availability of stroke services and hospital facilities at different hospital levels in Thailand: a cross-sectional survey study

**DOI:** 10.1186/s12913-022-08922-2

**Published:** 2022-12-20

**Authors:** Suthasinee Kumluang, Claudia Geue, Peter Langhorne, Olivia Wu

**Affiliations:** 1 Health Economics and Health Technology Assessment (HEHTA), School of Health and Wellbeing, University of Glasgow, Glasgow, UK; 2 School of Cardiovascular and Metabolic Health, University of Glasgow, Glasgow, UK

**Keywords:** Asia, Hospital, Hospital facilities, Stroke, Stroke service, Survey, Thrombolysis, Thailand

## Abstract

**Background:**

Stroke has one of the biggest burden of disease in Thailand and all health regions have been tasked to develop their service delivery to achieve the national key performance indicators set out by the Thai service plan strategy 2018–2022. Our aim was to characterise stroke services and hospital facilities by investigating differences in facilities across different hospital levels in Thailand.

**Methods:**

Self-complete questionnaires were distributed to 119 hospitals in 12 health regions between November–December 2019. Participants were health professionals whose main responsibilities are related to stroke service provision in their hospital. Descriptive statistics were used to report differences of stroke service provision between advanced-level, standard-level and mid-level referral hospitals.

**Results:**

Thirty-eight (32% response rate) completed questionnaires were returned. All advanced-level, standard-level (100%) and 55% of mid-level referral hospitals provided stroke units. Neurologists were available in advanced-level (100%) and standard-level referral hospitals (50%). Standard-level and mid-level referral hospitals only had a quarter of rehabilitation physicians compared to advanced-level referral hospital. Home-based rehabilitation was provided at 100% in mid-level but only at 16% and 50% in advanced-level and standard-level referral hospitals.

**Conclusions:**

Setting up a stroke unit, as a national goal that was set out in the service plan strategy 2018–2022, was achieved fully (100%) in advanced-level and standard-level referral hospitals including key essential supportive components. However, capacity in hospitals was found to be limited and stroke service delivery needs to be improved especially at mid-level referral hospitals. This should include regular organisational surveys and the use of electronic records to facilitate monitoring of clinical/health outcomes of patients.

**Supplementary Information:**

The online version contains supplementary material available at 10.1186/s12913-022-08922-2.

## Background

In Thailand, stroke is ranked second among the top ten causes of disability-adjusted life years [1] and stroke mortality rates (per 100,000 population) increased from 38.7 to 47.1 between 2014 and 2018 [2]. The stroke mortality rate in Thailand remains above the national key performance indicators (KPIs) [3]. To reduce the burden of stroke, the Thai ministry of public health (MOPH) developed national KPIs of stroke service delivery through their service plan strategy 2018–2022 according to hospital level.

The service plan strategy classifies the provinces in Thailand into 12 health regions (Additional file-Fig. S1). Each health region comprises of 4–8 provinces and serves 3–5 million people. Every health region has an authority to design their service delivery systems. As the MOPH considers the improvement of service provision in terms of health regions, hospitals in the same health region have to work together under the concept of a seamless health service network, self-contain and referral hospital cascade [3, 4]. Hospitals in all heath regions are grouped into different levels. A classification and numbers of referral hospitals is provided in supplementary materials (Additional file-Table S1 and Table S2).

Stroke care included stroke service delivery via the stroke fast track system which can include emergency medical services [5] or a ‘drip-and-ship’ model from spoke to hub hospital for thrombolytic treatment [6]. Spoke hospitals can be mid-level referral hospitals depending on capacity, while hub hospitals are mostly mid-level to advanced-level referral hospitals [7, 8]. For in-hospital care, thrombolysis treatment can be prescribed under supervision of a hub network. Some hospitals do not have a stroke unit (SU) but a stroke corner (SC) - a specialised area in in-patient wards, where healthcare professionals are able to provide more intensive treatments. Some hospitals provide in-patient rehabilitation services after the acute-phase or refer patients back to their contracted hospital for either outpatient rehabilitation or home visits. Village health volunteers (VHVs) from local communities play an important role in offering home visits as well as promoting primary health care [9, 10].

In terms of KPIs, the strategy stipulates that, in 2018, SU should be the standard of care and should be available in all advanced-level, and in 80% of standard-level referral hospitals within each health region [11]. All mid-level referral hospitals should establish rehabilitation units to reduce over-crowding of stroke care in advanced-level and standard-level referral hospitals and to increase capacity and accessibility of rehabilitation in rural areas [9, 10].

Following regular reviews, this was updated in 2019, and the service plan strategy stipulated that all advanced-level and standard-level referral hospitals have to set up SU while for mid-level referral hospitals this would be based on their performance (Table 1) [12].

**Table 1:** KPIs set by service plan, by hospital level

Level of care	Hospital level	No. of hospitals^a^	Stroke unit (SU) based-on service plan and goals within 5 years					
			2017^b^	2018	2019	2020	2021	2022
Tertiary care	Advanced-level referral hospitals	33	26	+ 7	–	–	–	–
	Standard-level referral hospitals	48	26	+ 5	+ 5	+ 5	+ 5	+ 2
	Mid-level referral hospitals	36	–	–	Setting up SU if hospitals have capacity.			

^a^data at year 2017; ^b^current number of hospitals providing stroke unit

Challenges in the provision of stroke services in Thailand remain, namely, a low rate of thrombolytic therapy with huge variation between health regions in terms of patterns of care and prescribing [13], limited stroke rehabilitation services in several regions, limited resources and infrastructure in provincial and rural areas [8, 14], A global observational study across 28 countries, included Thailand, (the INTERSTROKE study) [15] emphasised that poorer access to investigations, treatments, and services was found in low- and middle-income countries compared to high-income countries. Our aim was to characterise stroke services and hospital facilities by investigating differences in facilities across different hospital levels in Thailand. Findings from our study will help to address gaps in or barriers to stroke service provision and improve implementation and ultimately patient care.

## Methods

The study design was a cross-sectional survey study and reported according to the Checklist for Reporting Of Survey Studies (CROSS) (Additional file-Table S3).

### Questionnaire development

The questionnaire developed for this study (Additional File-Fig. S2) was adapted from the INTERSTROKE study [15] and contained six parts namely, hospital characteristics (e.g. hospital level, beds, number of staff), funding (e.g. sources of fund), SU characteristics (e.g. beds in SU, type and the number of staff providing care, proportion of stroke patients, multidisciplinary team meetings), other facilities and services related to stroke services (e.g. their own written guidance, type of clinical assessment scores, systems of complication prevention), post-stroke care (e.g. type of rehabilitation provided, type of staff), and suggestions and feedback for services improvement.

Before circulating the questionnaire, it was reviewed by two non-academic healthcare staff from hospitals (neurologists) and one academic staff member. After revision, a pilot questionnaire was sent to four secondary hospitals.

### Study sites and participants

Questionnaires were distributed to 119 hospitals under the MOPH service plan strategy which does not include hospitals within Bangkok and other university hospitals (one questionnaire per hospital). Hospitals are classified into: (1) 34 advanced-level, (2) 49 standard-level and (3) 36 mid-level referral hospitals. Questionnaires were sent to health professionals whose main responsibilities are related to stroke service provision in their hospital.

### Ethics approval

Ethical approval was sought by the first author and granted by the Ethical Review Committee of the Institute for Development of Human Research Protection, MOPH (No.IHRP722/2562). All research was performed in accordance with the declaration of Helsinki ethical principles. Ethical approval was granted for public hospitals under the Thai MOPH (12 health regions).

### Data collection

The questionnaire was distributed via post, online and e-mail (pdf file) to all hospitals between November–December 2019 (Additional file-Table S4). All hospitals received the questionnaire via post with a link to the online questionnaire. Telephone calls were offered if clarification was required by respondents. A follow-up email was sent 4 weeks after distribution, followed by telephone reminders every 2 weeks. The survey was closed at month seven. Data were collected for the fiscal year 2018 (1 October 2017–30 September 2018). Data were entered or exported into Microsoft Excel spreadsheets before being transferred to R for analysis.

### Statistical analysis

Data were analysed by comparing service availability, healthcare staffing, stroke service characteristics and rehabilitation. Comparisons were made between hospital levels. All analyses were undertaken using R software. Descriptive statistics were used to compare differences in the services provided.

## Results

We received responses from 38 hospitals (response rate 32%). The majority was returned by post (66%, *N* = 25), followed by online questionnaires (24%, *N* = 9) and email (11%, *N* = 4). Respondents who provided the information were nurses (98%, *N* = 37) and physicians (2%, *N* = 1). Eight (21%) of these were advanced-level, 19 (50%) standard-level and 11 (29%) mid-level referral hospitals, representing all health regions except regions 7 and 13 (Bangkok). The highest proportion of respondents (18%) came from health regions 5 and 11 (Additional file-Fig. S3 and Fig. S4).

### Hospital characteristics

The number of dedicated stroke beds (Table 2) was similar at each hospital level. Eight (1.1%, *N* = 705), six (1.5%, *N* = 400) and four beds (1.6%, *n* = 252) were dedicated to stroke patients in advanced-level, standard-level and mid-level referral hospitals, respectively.

**Table 2:** Hospital characteristics

	Advanced-level referral hospitals		Standard-level referral hospitals		Mid-level referral hospitals	
	Median (IQR)	N	Median (IQR)	N	Median (IQR)	N
**Number of beds**						
Total beds	705 (165)	8	400 (189)	19	252 (74)	11
Stroke beds	8 (4)	8	6 (4)	19	4 (3)	10
**Number of stroke admissions per year**						
	**Median (IQR); percentage**	**N**	**Median (IQR); percentage**	**N**	**Median (IQR); percentage**	**N**
Ischemic	984 (721); 63%	8	438 (244); 62%	19	408 (194); 78%	11
Haemorrhagic	538 (211); 33%	8	215 (162); 25%	19	62 (68); 19%	11
Unspecified	77 (158); 4%	8	123 (157); 13%	19	8 (8); 3%	11
**Number of transfers to other hospitals per year**						
Ischemic	63 (69); 42%	3	6 (9); 47%	16	25 (29); 75%	11
Haemorrhagic	84 (20); 51%	3	15 (16); 29%	16	12 (19); 18%	11
Unspecified	12 (9); 7%	3	9 (7); 24%	16	2 (8); 7%	11
**Number of physician**^a^						
	**Median (IQR)**	**N**	**Median (IQR)**	**N**	**Median (IQR)**	**N**
General practice	7 (14)	7	3 (15)	12	6 (9)	6
Internal medicine	20 (21)	8	9 (8)	19	5 (2)	11
Neurologist	2 (1)	8	1 (1)	8	–	–
Surgeon	11 (10)	7	5 (4)	19	3 (1)	11
Neurosurgeon	3 (1)	8	2 (1)	13	1 (−)	2
Emergency medicine	5 (3)	7	2 (3)	15	2 (1)	8
Radiologist	5 (2)	7	2 (3)	19	2 (1)	9
Rehabilitation physician	4 (2)	8	1 (1)	17	1 (−)	5
Family medicine	3 (2)	7	2 (1)	16	3 (2)	9
**Proportion of stroke patients who are looked after by a specialist doctors training in stroke**						
median	80 (42)	8	80 (40)	12	100 (43)	3
**Proportion of stroke fast track (SFT) network and/or referral system with other hospitals**						
**SFT network**						
SFT - N (%)	2 (25)	8	13 (68)	19	9 (82)	11
SFT hub - N (%)	5 (63)	8	4 (21)	19	0	11
**Referral system**						
refer - N (%)	3 (37)	8	3 (37)	19	11 (100)	11
referral hub - N (%)	5 (63)	8	5 (63)	19	0	11

*N* Number of hospitals, *IQR* Inter quartile range

^a^this number represents the number of staff in hospitals which might not have a role in stroke service delivery

In terms of number of patient admissions and referrals, a higher mean percentage of stroke patient admissions was observed for ischemic stroke for all hospital levels. However, haemorrhagic stroke patient admissions in advanced-level hospitals was 1.7 times higher than in mid-level referral hospitals. In addition, mainly patients with ischemic stroke were transferred to other hospitals (standard-level 47%, mid-level 75%). In contrast, mainly patients with haemorrhagic stroke were transferred from advanced-level referral hospitals (51%).

The main type of physician at all three hospital levels was similar, including general practice, internal medicine and surgeons. Neurologists were available in advanced-level (100%) and standard-level (42%), but not in mid-level referral hospitals. Rehabilitation physicians were available in all advanced-level and standard-level, and in 50% of mid-level referral hospitals.

Additionally, the median percentage of stroke patients who were reported to be looked after by specialist doctors with training in stroke was highest in mid-level referral hospital (advanced-level: 80%, standard-level: 80%, mid-level: 100%). However, 10 hospitals (three standard-level and seven mid-level referral hospitals) reported 0% as they did not have a specialist in their hospitals.

Lastly, five out of eight (63%) advanced-level referral hospitals served as a stroke-fast-track hub for lower-level hospitals, while only four (21%) standard-level referral hospitals had the capacity to be a hub/node for mid-level referral hospitals.

### Healthcare service funding

The funding sources did not differ between hospitals (*N* = 34), with most hospitals reporting that nearly 100% of funding came from government. There is generally no requirement for out-of-pocket payments from patients.

### Stroke unit (SU) services

Overall, 33 hospitals (87%) reported having SUs (Table 3). This included all advanced-level and standard-level and six mid-level (54%) referral hospitals. While four mid-level referral hospitals reported to provide SC. Only one mid-level referral hospitals reported not to have either SU or SC.

**Table 3:** Stoke unit services

	Advanced-level referral hospitals		Standard-level referral hospitals		Mid-level referral hospitals	
	percentage	N	percentage	N	percentage	N
Able to provide stroke unit (SU)	100	8	100	19	54	11
Percentage of patients admitted to SU/stroke corner (SC)^a^	64	8	67	19	77	8
Supportive features of SU/SC						
1) Written standard protocols	100	8	95	19	90	10
2) Discrete ward area	100	8	89	19	100	10
3) Staff whose work is mainly managing stroke patients	100	8	100	19	90	10
4) Multidisciplinary team	100	8	100	19	90	10
5) Special education programmes for staff	75	8	95	19	100	10
6) Information and education for stroke patients/caregivers	100	8	100	19	100	10
Type and number of staff providing care in SU/SC						
	**median (IQR)**	**N**	**median (IQR)**	**N**	**median (IQR)**	**N**
Internal medicine	4 (5)	3	2 (4)	14	4 (3)	10
Neurology	2 (2)	6	1 (−)	5	–	–
Neurosurgery	3 (1)	6	1 (−)	8	1 (−)	1
Radiology	5 (4)	3	2 (1)	8	2 (1)	7
Rehabilitation physician	2 (2)	5	1 (−)	13	1 (−)	4
Nurse	13 (8)	6	8 (6)	16	8 (5)	9
Physiotherapist	1 (1)	6	1 (2)	14	4 (3)	10
Social worker	1 (−)	4	1 (−)	11	1 (−)	9
Multidisciplinary team meetings						
	**n (%)**	**N**	**n (%)**	**N**	**n (%)**	**N**
Once per day	2 (25)	8	6 (32)	18	1 (11)	11
Once per week	3 (38)	8	3 (16)	18	–	11
Less than once per week	3 (38)	8	9 (47)	18	9 (82)	11
- twice a month	–	3	1 (11)	9	1 (11)	9
- monthly	1 (33)	3	4 (44)	9	1 (11)	9
- two or three monthly	1 (33)	3	2 (22)	9	4 (44)	9
- not stated	1 (33)	3	2 (22)	9	3 (33)	9
No multidisciplinary team	–	8	–	18	1 (11)	11
Staff delivering rehabilitation						
Doctor	4 (50)	8	9 (47)	19	6 (55)	11
Nurse	7 (88)	8	1 (84)	19	8 (73)	11
Physiotherapist	8 (100)	8	1 (95)	19	1 (10)	11
Occupational therapist	6 (75)	8	9 (47)	19	2 (18)	11
Speech therapist	1 (13)	8	3 (16)	19	–	11
Psychologist	2 (25)	8	5 (26)	19	2 (18)	11
Village health volunteer	5 (63)	8	11 (58)	19	8 (73)	11

*n* Number of hospitals providing services, *N* Number of hospitals, *IQR* Inter quartile range

^a^Divided by total number of stroke patients

The proportion of stroke patients admitted to SU/SC was evaluated. The highest average percentage reported was found in mid-level referral hospitals at 77% (*N* = 8), while in advanced-level and standard-level referral hospitals proportions were 64% (*N* = 8), and 67% (*N* = 19), respectively. However, there were wide variations between health regions in advanced-level referral hospitals with four hospitals reporting that the proportion of stroke patients admitted to SU/SC was ≤50%. Similar proportions were reported at four standard-level and one mid-level referral hospitals.

Results of supportive features for SU/SC showed that 28 hospitals (76%) were able to provide all six features. All features were provided by all advanced-level referral hospitals except for special education programmes for staff (75%). Only three features were provided at all standard-level (feature 3, 4 and 6) and mid-level referral hospitals (feature 2, 5 and 6). In addition, 75% of advanced-level, 79% of standard-level, and 33% of mid-level referral hospitals passed the criteria for stroke centre certification recommended by Thai MOPH.

Neurologists, neurosurgeons, rehabilitation physicians, nurses, physiotherapists and occupational therapists were usually involved in providing care in SU/SC in all advanced-level referral hospitals. In standard-level referral hospitals, the main type of staff was similar but the number of staff was lower compared to advanced-level referral hospitals. In mid-level referral hospitals, there was no neurologists at SU/SC and the number of overall staff being lower except for physiotherapists. The type and number of staff providing care in SU/SC, either full-time or part-time is presented in Additional file-Table S5.

All advanced-level and standard-level referral hospitals reported to provide rehabilitation in SU/SC, but only 73% in mid-level referral hospitals. For all hospital levels, the key staff delivering rehabilitation after patients were discharged from hospital comprised physiotherapists, nurses, occupational therapists and VHVs. Most VHV provided rehabilitation services in mid-level referral hospitals.

### Other healthcare facilities and services to support stroke service provision

The main types of clinical assessment (Table 4) included the Glasgow coma scale, Barthel Index (BI) and swallowing impairment at all hospital levels. Not all standard-level and mid-level referral hospitals used the modified Rankin Score (mRS) at admission. The type of assessment differed in standard-level referral hospitals with some noticeable variations in using the mRS with one hospital not conducting mRS. BI assessment was conducted at all hospital levels at admission, however, not all standard-level and mid-level referral hospitals measured BI at discharge. A less frequent test was malnutrition.

**Table 4:** Clinical assessment at admission or discharge from hospital

Clinical assessment	Advanced-level referral hospitals (***N*** = 8)		Standard-level referral hospitals (***N*** = 18)		Mid-level referral hospitals (***N*** = 11)	
	Admission	Discharge	Admission	Discharge	Admission	Discharge
Glasgow coma scales	100%	100%	100%	89%	100%	82%
Barthel Index	100%	100%	100%	89%	100%	91%
modified Rankin Score	100%	100%	72%	78%	82%	82%
National Institutes of Health Stroke Scale	100%	100%	94%	67%	82%	73%
Swallowing impairment	100%	100%	100%	61%	100%	82%
Level of consciousness	100%	100%	94%	78%	91%	82%
Malnutrition	50%	50%	78%	50%	73%	36%

The proportion of thrombolytic therapy (Table 5) was less than 10%. There were notable differences in the proportion of prescribing thrombolysis between hospitals within the same health region at all hospital levels. It should be noted that two mid-level referral hospitals did not provide thrombolysis although they provided SC. Furthermore, 58% (*N* = 22) of hospitals provided only two investigations within 24 hours after admission including electrocardiogram monitoring and computerized tomography (CT) of the brain.

**Table 5:** Proportion of patients receiving thrombolysis and proportion of hospitals providing investigations after admission

	Advanced-level referral hospitals	N	Standard-level referral hospitals	N	Mid-level referral hospitals	N
Recombinant tissue plasminogen activator drug						
Average	8.1%	7	10.6%	18	8.8%	8
Antiplatelet drugs						
Median (IQR)	99 (19)	8	94 (13)	17	92 (14)	10
Min-max	64–100		71–100		10–100	
Electrocardiogram monitor						
Median (IQR)	100 (−)	8	98 (48)	18	100 (2)	11
Min-max	99–100		6–100		80–100	
Computerized tomography brain						
Median (IQR)	100 (−)	8	100 (−)	19	100 (−)	11
Min-max	99–100		6–100		80–10	
Magnetic resonance imaging/angiography						
Median (IQR)	20 (78)	6	2 (2)	3	1 (5)	3
Min-max	1–100		1–5		1–10	
Carotid doppler ultrasound						
Median (IQR)	30 (20)	2	10 (30)	3	5 (49)	4
Min-max	10–50		2–62		1–98	

*N* Number of hospitals, *IQR* Inter quartile range

### Post-stroke care services

There was considerable variation in the proportion of stroke patients receiving rehabilitation (Table 6). This was found for all types of services available and was not only observed between health regions but also between different hospital levels, and within hospital levels.

**Table 6:** Type of rehabilitation and percentage of hospital providing rehabilitation

	Advanced-level referral hospitals	N	Standard-level referral hospitals	N	Mid-level referral hospitals	N
A.Rehabilitation						
Inpatient rehabilitation (*N* = 31)						
Median (IQR)	100 (16)	7	97 (12)	15	98 (5)	9
Min-max	72–100		40–100		92–100	
Inpatient, then outpatient rehabilitation (*N* = 27)						
Median (IQR)	83 (25)	6	50 (67)	13	93 (21)	8
Min-max	30–100		5–100		20–100	
Outpatient rehabilitation (*N* = 23)						
Median (IQR)	20 (40)	5	48 (70)	12	91 (67)	6
Min-max	15–100		5–100		9–100	
Home-based rehabilitation (*N* = 26)						
Median (IQR)	16 (62)	5	50 (79)	12	100 (15)	9
Min-max	6–74		5–100		55–100	
Community-based rehabilitation (*N* = 18)						
Median (IQR)	26 (21)	3	20 (83)	9	92 (41)	6
Min-max	3–46		5–100		10–100	
B.Intermediate care (IMC)						
Proportion of hospitals providing IMC services-N (%)	7 (88)	8	18 (95)	19	8 (73)	11
C.Long-term care (LTC)						
Proportion of hospitals providing LTC services-N (%)	7 (88)	8	12 (75)	16	4 (44)	9

*N* Number of hospitals, *IQR* Inter quartile range

### Suggestions for stroke service improvement from respondents

Respondents were also asked to provide comments with regards to improving stroke services. In advanced-level referral hospitals, these suggestions comprised: a clear policy and direction in order to respond to the MOPH policy, government funding to support human resource development, and an efficient system of health data records and linkage between hospitals along with monitoring and data analysis teams. Staff from standard-level and mid-level referral hospitals, commented on the need for materials and facilities to be able to care for patients at their own home and the need for a budget and manpower to be able to provide appropriate care post-discharge. Moreover, staff at some mid-level referral hospitals reported they would require ongoing teaching support from the higher-level hospitals to improve patient care in SUs.

## Discussion

This is the first study to provide a detailed picture of healthcare facilities and services available to stroke patients in Thailand. The number of dedicated beds for stroke patients was similar between health regions at all hospital levels. In terms of KPIs from MOPH inspection report, stroke mortality in fiscal year 2019 and 2020 decreased in some health regions, although the national stroke mortality rate remains at 8% (2019: 7.97% and 2020: 7.99%), not reaching the national target of less than 7% per year [16]. Many patients were transferred from advanced-level referral hospitals, this result may be explained by the fact that patients might need to receive specialist care during the acute phase, such as surgery for haemorrhagic stroke. Patients are referred back to their registered hospitals when they are clinically stable.

The available specialties were different between hospital levels, especially neurologists were available in advanced-level and standard-level referral hospitals due to the scarcity of neurologists. The service plan strategy [3] sets out that, at SU, at least one neurologist and/or neurosurgeon and/or internal medicine practitioner should be available as a minimum standard requirement. The availability of these specialists therefore very much determines whether standard-level referral hospitals are able to provide SUs. Moreover, approximately 50% of neurologists practice in the Bangkok area [17] with the remaining neurologists ranging from 3 to 27 per health region which could contribute to the shortage of professionals in rural areas [5, 10]. Nurses were the main group providing stroke care at all hospital levels. The service plan strategy indicates that at least four nurses should be available at SUs as a minimum standard requirement, and results from our survey confirmed an adequate number of nursing staff.

Our findings also highlight that all advanced-level and standard-level referral hospitals had a SU, thus achieving the goal set by the service plan strategy (100% at advanced-level and standard-level referral hospitals). Some mid-level referral hospitals were also able to set up SU/SC. Despite some hospitals with SU being eligible to apply for stroke centre certification, not all have had the certification. The stroke centre certification assessment was recommended by the Department of Medical Services, MOPH, Thailand. They recommended that hospitals should improve SU and should obtain certification to provide standard of stroke care for acute stroke patients and hospitals should be accredited or re-accredited every 3 years [18]. In addition to the lowest percentage of access to SU in advanced-level referral hospitals, we found that one hospital reported their SU had just been set up a few months before answering our questionnaire; thus, the percentage of access to SU at this hospital was less than 30%. Although, the percentage of access to SU was almost 70%, the percentage of stroke patients admitted to SU/SC was quite low compared to some developed countries [19, 20].

Furthermore, this study supports evidence from the INTERSTROKE study that CT scans and antiplatelet drugs given were nearly 100%, while provision of MRI scans, carotid doppler ultrasound and thrombolytic therapy was extremely low.

The type and number of staff involved in providing care in SU/SC differed between hospital levels. One possible explanation could be the limited number of staff based at these hospitals and the scarcity of specialists in standard-level referral hospitals [5]. This is consistent with previous studies [21, 22] indicating that mid-level not only had fewer health professionals than advanced-level or standard-level referral hospitals but also that there was maldistribution of healthcare workers.

Furthermore, our finding shows that standard-level had greater provision of thrombolysis compared to advanced-level referral hospitals. It seems possible that stroke service delivery was improved due to the improvement of stroke fast track and thrombolytic treatment in mid-level and standard-level referral hospitals. Thrombolysis treatment can be provided at mid-level referral hospitals by non-neurologist, such as internists or emergency physicians, under the supervision of a neurologist from advanced-level or standard-level referral hospitals [23]. It should be noted that there could be regional differences which ranging from 5 to 22%. These results are supported by a Thai study using national stroke data [23] which showed that the percentage of acute ischemic stroke patients who were treated with thrombolysis varied widely across the country.

Although, there is no agreed benchmark for thrombolysis rates, the utilization rate remains low compared to other countries, especially developed countries [24–26]. Possible explanations include the onset of symptoms was more than 4.5 hours [5, 14], or patients may have had contraindications or a poor prognosis, which could affect the rate of thrombolysis initiation.

One interesting finding relates to the clinical assessment scores. Not all hospitals, especially standard-level and mid-level referral hospitals, evaluated patients at both admission and discharge. While the intermediate care guidelines [27], focussing on the recovery phase, recommended to measure the need for rehabilitation using the BI scores. This will affect health outcome assessments in the post-acute period. Further, the proportion and type of rehabilitation differed between hospital levels. Advanced-level and standard-level referral hospitals focused on inpatient rehabilitation, while most home-based and community rehabilitation were provided by mid-level referral hospitals. Indeed, these findings reflect the real-world service delivery in Thailand with the higher-level hospitals having the capacity to provide specialised care, while lower-level hospitals having fewer resources offering basic care [9, 10],

Thailand has introduced VHVs [9, 10] based in the community and VHVs play an important role in supporting healthcare providers such as follow-up care and acting as a link between providers and community resources. A previous study indicated that well-trained VHVs could help improve quality of life for stroke survivors [21]. However, VHVs seem to be unique to a Thai context and no international comparison can be made here.

Our study offers novel insights into stroke care in Thailand. Our questionnaire was adapted from the INTERSTROKE study [15], allowing us to draw comparisons. Despite low response rates, we were able to include representatives of almost all geographical areas in Thailand. Our results seem consistent with the INTERSTROKE study but should be interpreted with caution. Some limitations arise due to the nature of the survey method. Our study does not cover all hospital types, such as Bangkok area and university hospitals which mostly serve as excellence centres. This will have led to limitations in terms of comparability between health regions. Another limitation arises due to a lack of information on secondary prevention medication.

Moving forward we suggest that the stroke organisational survey should be reviewed and updated regularly in the MOPH annual reports and audit systems. The ability to track changes over time in stroke service quality should be the cornerstone of stroke provision. The use of patient-level data could help to improve information that is fed back to health facilities. Developing a system of national health data records and linkage between hospitals would be valuable for collecting data on clinical assessments and continuous stroke care between hospitals. Moreover, the national data should not be a fragmented database, rather, it should be in co-operation between heath schemes and MOPH.

## Conclusions

The present study highlights that hospitals at all levels are likely to have shown improvement in service delivery, achieving the goals set by the service plan strategy in terms of setting up SUs with essential supportive features. Although, mid-level referral hospitals have potential to provide stoke service delivery similar to standard-level or advanced-level referral hospitals, improvements still need to be made in areas of health care workforce. Data linkage and health record systems for clinical or health outcomes in order to follow-up and monitor health outcomes of patients should be developed between hospitals and at national levels. Further, the limited capacity of service delivery should encourage policy makers to further improve stroke care at mid-level referral hospitals in Thailand.

## Supplementary Information

**Additional file 1: Fig. S1.** Map of health regions in Thailand. **Table S1.** Classification of referral hospitals and goal of stroke service delivery under Thai service plan strategy [1–3]. **Table S2.** Number of hospitals by health region at year 2019 [4]. **Table S3.** Checklist for Reporting Of Survey Studies (CROSS). Questionnaire administration. **Fig. S2.** Stroke questionnaire adapted from the INTERSTROKE study. **Table S4**. Data collection timeline. **Fig. S3.** Number of questionnaires received and proportion of questionnaires (*N* = 38). **Fig. S4.** Response rate, by health region (*N* = 38). **Table S5.** Type and number of staff providing care for stroke patients in stroke unit/stroke corner.

## Data Availability

The data that support the findings of this study are available upon request from the corresponding author, [SK]. The data are not publicly available due to containing information that could compromise the privacy of research participants. Data are available from the authors upon reasonable request and with permission of each hospital that may require an additional document.

## Abbreviations

BI: Barthel index
CT: Computerized tomography
KPIs: Key performance indicators
mRS: Modified Rankin Score
MOPH: Ministry of public health
SC: Stroke corner
SU: Stroke unit
VHV: Village health volunteers
