# Supplementary material for: Availability of stroke services and hospital facilities at different hospital levels in Thailand: a cross-sectional survey study
Source: BMC Health Serv Res. 2022 Dec 20;22:1558. doi: 10.1186/s12913-022-08922-2 (PMC9764597; doi:10.1186/s12913-022-08922-2)
Supplement: Supplementary file 1 — Additional file 1: Fig. S1. Map of health regions in Thailand. Table S1. Classification of referral hospitals and goal of stroke service delivery under Thai service plan strategy [1–3]. Table S2. Number of hospitals by health region at year 2019 [4]. Table S3. Checklist for Reporting Of Survey Studies (CROSS). Questionnaire administration. Fig. S2. Stroke questionnaire adapted from the INTERSTROKE study. Table S4. Data collection timeline. Fig. S3. Number of questionnaires received and proportion of questionnaires (N = 38). Fig. S4. Response rate, by health region (N = 38). Table S5. Type and number of staff providing care for stroke patients in stroke unit/stroke corner. [file 12913_2022_8922_MOESM1_ESM.pdf]

Additional file

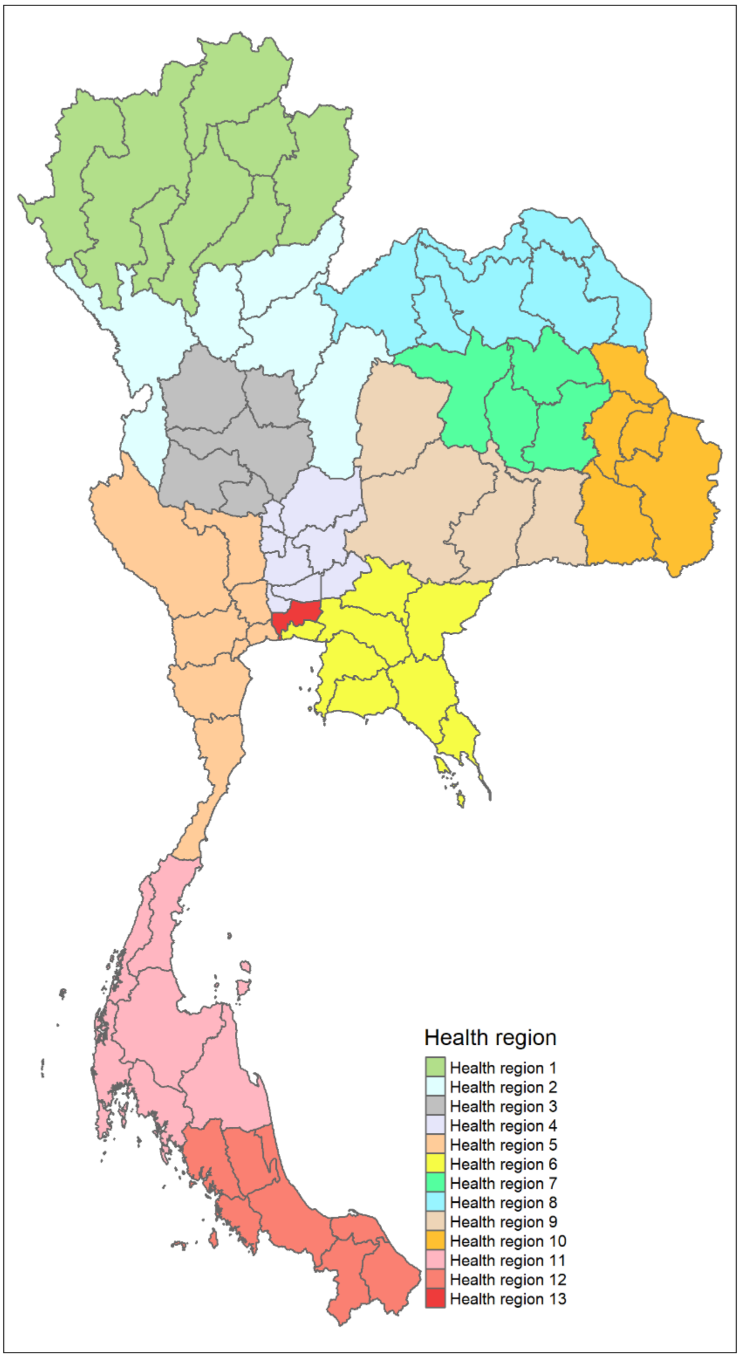

Figure S1 Map of health regions in Thailand

Table S1 Classification of referral hospitals and goal of stroke service delivery under Thai service plan strategy [1-3].

Referral hospital level[1]	Hospital level	Level of care	Number of hospitals	Details	Stroke service based-on service plan and goals within 5 years					
					2017	2018	2019	2020	2021	2022
High-level referral hospitals	Advanced-level referral hospitals (A-level)	Tertiary care	33	• Number of beds: more than 500 beds<br>• All major medical specialists, all minor specialties and all sub-specialties.<br>• It can deliver advance and sophisticate health technology and can have a function as the medical school.<br>• It is able to support referral patients from regional/general hospitals[2] within the district or nearby areas covering 4-8 provinces per one advance-level referral hospital.	26	7	-	-	-	-
High-level referral hospitals	Standard-level referral hospitals (S-level)	Tertiary care	48	• Number of beds: more than 300 - 500 beds.<br>• It is a provincial general hospital and designated as the node of the provincial network.<br>• The hospital has medical specialists in all major specialties, all minor specialties and sub - specialties in some fields (as needed).<br>• It is able to support referral patients in a provincial service network[3].	26	5	5	5	5	2
Mid-level	Mid-level referral	Tertiary care	36	• Number of beds: more than 120 - 300 beds.	-	-	Setting up stroke unit if hospitals have capacity.			

Referral hospital level[1]	Hospital level	Level of care	Number of hospitals	Details	Stroke service based-on service plan and goals within 5 years					
					2017	2018	2019	2020	2021	2022
referral hospitals	hospitals (M1-level)			• It is a district general hospital having medical specialists in all 6 major specialties (physician, surgeon, obstetrician, paediatrician, orthopaedist and anaesthesiologist) and some minor specialties that are required.<br>• It is designated as a referral hospital for patients from the secondary care in their service network.						
Mid-level referral hospitals	Mid-level referral hospitals (M2-level)	Secondary care	88	• Number of beds: more than 120 - 300 beds.<br>• It is a big/node community hospitals having medical specialists in all 6 major specialties (physician, surgeon, obstetrician, paediatrician, orthopaedist and anaesthesiologist)<br>• It is designated as a referral hospital for patients from the secondary care in first-level referral hospitals.	-	-	-	-	-	-
First-level referral hospital	First-level referral hospital (F-level)	Secondary care	694	• Number of beds: from 10 to more than 120 beds.<br>• It is a district hospital or community hospital providing the services that cover basic primary health care and secondary cares which mean that they have clinical capacity to provide admission services.	-	-	-	-	-	-

Referral hospital level[1]	Hospital level	Level of care	Number of hospitals	Details	Stroke service based-on service plan and goals within 5 years					
					2017	2018	2019	2020	2021	2022
				• They are able to provide treatments from medical specialties in all or some fields (major specialty: obstetrics, orthopaedics, paediatrics, surgery, and medicine)						
N/A	Primary health care	Primary care	N/A	• The primary health care are the smallest size and offer basic primary care services for several type of health prevention and promotion services, but not for admission services.<br>• They are closed to the community, village or patient's home and the distance is usually less than 30 minutes from home to primary care unit.	-	-	-	-	-	-

[1]classification based on service plan strategy
[2]regional/general hospitals served as tertiary hospital level and mostly located in big provincial cities throughout Thailand.
[3]provincial service network defined as a network between provinces such as a seamless services for stroke fast track.

Table S2 Number of hospitals by health region at year 2019 [4]

Health region	Advanced-level referral hospitals	Standard-level referral hospitals	Mid-level referral hospitals (M1-level)	Mid-level referral hospitals (M2-level)	First-level referral hospital	Total
1	3	5	3	8	83	102
2	2	4	1	6	34	47
3	1	4		5	44	54
4	3	5	4	6	53	71
5	4	6	5	6	45	66
6	6	3	5	5	54	73
7	2	2	2	12	59	77
8	2	5	3	5	73	88
9	3	2	4	13	67	89
10	2	4	3	4	58	71
11	3	4	4	10	60	81
12	3	5	2	4	64	78
**Total**	**34**	**49**	**36**	**84**	**694**	**897**

Table S3 Checklist for Reporting Of Survey Studies (CROSS)

Section/topic	Item	Item description	Reported on page #
**Title and abstract**			
Title and abstract	1a	State the word "survey" along with a commonly used term in title or abstract to introduce the study's design.	1
	1b	Provide an informative summary in the abstract, covering background, objectives, methods, findings/results, interpretation/discussion, and conclusions.	1-2
**Introduction**			
Background	2	Provide a background about the rationale of study, what has been previously done, and why this survey is needed.	2-4
Purpose/aim	3	Identify specific purposes, aims, goals, or objectives of the study.	4
**Methods**			
Study design	4	Specify the study design in the methods section with a commonly used term (e.g., cross-sectional or longitudinal).	5
	5a	Describe the questionnaire (e.g., number of sections, number of questions, number and names of instruments used).	5
Data collection methods	5b	Describe all questionnaire instruments that were used in the survey to measure particular concepts. Report target population, reported validity and reliability information, scoring/classification procedure, and reference links (if any).	5
	5c	Provide information on pretesting of the questionnaire, if performed (in the article or in an online supplement). Report the method of pretesting, number of times questionnaire was pre-tested, number and demographics of participants used for pretesting, and the level of similarity of demographics between pre-testing participants and sample population.	5
	5d	Questionnaire if possible, should be fully provided (in the article, or as appendices or as an online supplement).	Additional file 2.
Sample characteristics	6a	Describe the study population (i.e., background, locations, eligibility criteria for participant inclusion in survey, exclusion criteria).	5
	6b	Describe the sampling techniques used (e.g., single stage or multistage sampling, simple random sampling, stratified sampling, cluster sampling, convenience sampling). Specify the locations of sample participants whenever clustered sampling was applied.	5
	6c	Provide information on sample size, along with details of sample size calculation.	5
	6d	Describe how representative the sample is of the study population (or target population if possible), particularly for population-based surveys.	5
Survey administration	7a	Provide information on modes of questionnaire administration, including the type and number of contacts, the location where the survey was conducted (e.g., outpatient room or by use of online tools, such as SurveyMonkey).	5-6
	7b	Provide information of survey's time frame, such as periods of recruitment, exposure, and follow-up days.	6 and additional file 2.
	7c	Provide information on the entry process:<br>–>For non-web-based surveys, provide approaches to minimize human error in data entry.<br>–>For web-based surveys, provide approaches to prevent "multiple participation" of participants.	5-6
Study preparation	8	Describe any preparation process before conducting the survey (e.g., interviewers' training process, advertising the survey).	5-6

Section/topic	Item	Item description	Reported on page #
Ethical considerations	9a	Provide information on ethical approval for the survey if obtained, including informed consent, institutional review board [IRB] approval, Helsinki declaration, and good clinical practice [GCP] declaration (as appropriate).	6 and additional file 2
	9b	Provide information about survey anonymity and confidentiality and describe what mechanisms were used to protect unauthorized access.	additional file 2
Statistical analysis	10a	Describe statistical methods and analytical approach. Report the statistical software that was used for data analysis.	6
	10b	Report any modification of variables used in the analysis, along with reference (if available).	n/a
	10c	Report details about how missing data was handled. Include rate of missing items, missing data mechanism (i.e., missing completely at random [MCAR], missing at random [MAR] or missing not at random [MNAR]) and methods used to deal with missing data (e.g., multiple imputation).	n/a
	10d	State how non-response error was addressed.	n/a
	10e	For longitudinal surveys, state how loss to follow-up was addressed.	n/a
	10f	Indicate whether any methods such as weighting of items or propensity scores have been used to adjust for non-representativeness of the sample.	n/a
	10g	Describe any sensitivity analysis conducted.	n/a
**Results**			
Respondent characteristics	11a	Report numbers of individuals at each stage of the study. Consider using a flow diagram, if possible.	6
	11b	Provide reasons for non-participation at each stage, if possible.	n/a
	11c	Report response rate, present the definition of response rate or the formula used to calculate response rate.	6
	11d	Provide information to define how unique visitors are determined. Report number of unique visitors along with relevant proportions (e.g., view proportion, participation proportion, completion proportion).	n/a
Descriptive results	12	Provide characteristics of study participants, as well as information on potential confounders and assessed outcomes.	6
Main findings	13a	Give unadjusted estimates and, if applicable, confounder-adjusted estimates along with 95% confidence intervals and p-values.	n/a
	13b	For multivariable analysis, provide information on the model building process, model fit statistics, and model assumptions (as appropriate).	n/a
	13c	Provide details about any sensitivity analysis performed. If there are considerable amount of missing data, report sensitivity analyses comparing the results of complete cases with that of the imputed dataset (if possible).	n/a
**Discussion**			
Limitations	14	Discuss the limitations of the study, considering sources of potential biases and imprecisions, such as non-representativeness of sample, study design, important uncontrolled confounders.	17
Interpretations	15	Give a cautious overall interpretation of results, based on potential biases and imprecisions and suggest areas for future research.	17
Generalizability	16	Discuss the external validity of the results.	17
**Other sections**			

Section/topic	Item	Item description	Reported on page #
Role of funding source	17	State whether any funding organization has had any roles in the survey's design, implementation, and analysis.	18
Conflict of interest	18	Declare any potential conflict of interest.	18
Acknowledgements	19	Provide names of organizations/persons that are acknowledged along with their contribution to the research.	19

Questionnaire administration

1. Questionnaire development

The service level data of each hospital were collected using the developed questionnaires. The INTERSTROKE questionnaire [5] was adapted by all authors (PL was an author for the INTERSTROKE questionnaire) before translating to Thai language. Adaptations were made to reflect the Thai context.

Questionnaire contained six parts as namely (1) hospital characteristics (e.g. hospital level, beds, number of staff), (2) healthcare service funding (e.g. sources of fund), (3) stroke unit characteristics (e.g. beds in stroke unit, type and the number of staff providing care, proportion of stroke patients, multidisciplinary team meetings), (4) other facilities and services related to stroke services (e.g. their own written guidance, type of clinical assessment scores, systems of complication prevention), (5) post-stroke care (e.g. type of rehabilitation provided, type of staff) and (6) in suggestions and feedback for stroke service delivery improvement.

2. Stroke questionnaire adapted from the INTERSTROKE study

It should be noted that this questionnaire version (Figure S2) has been adapted to Thai language. The Thai questionnaire version was submitted and granted by the Ethical Review Committee of the Institute for Development of Human Research Protection, MOPH (No.IHRP722/2562) prior to conducting this study.

HiTAP HeHTA Questionnaire ID: ☐☐☐☐☐

Stroke service questionnaire

We would like to collect the description of the type of service and care that the majority of stroke patients will receive at your centre. Please put **X** mark in the box ☐ in front of the statement and/or fill in the blank truthfully. If there is no service available, please enter/fill 0.

A. Hospital Characteristics			
A1	Province		
A2	Hospital name		
A3	Hospital level	☐ A ☐ S ☐ M1 ☐ Other (specify)	
A4	Area health		
A5	How many beds are in your hospital?	In total<br>In medicine departments (i.e. non-surgery)<br>Dedicated for stroke patients	☐<br>☐<br>☐
A6	How many physicians are in your hospital?	In total<br>General practice<br>Internal medicine<br>Neurologist<br>Certificate in Fellowship Training in Stroke<br>Surgeon<br>Neurosurgeon<br>Emergency medicine<br>Radiologist/ Interventional radiologist<br>Rehabilitation physician<br>Family medicine<br>Other (specify) ______	☐<br>☐<br>☐<br>☐<br>☐<br>☐<br>☐<br>☐<br>☐<br>☐<br>☐
A7	On average, how many stroke patients are admitted to your hospital each year?	In total (ICD10: I60-I69)<br>Ischemic stroke (ICD10: I63)<br>Haemorrhagic stroke (ICD10: I60-I62)<br>Unspecified stroke (ICD10: I64-I69)	☐<br>☐<br>☐<br>☐
A8	On average, how many stroke patients are transferred to other hospitals each year?	In total (ICD10: I60-I69)<br>Ischemic stroke (ICD10: I63)<br>Haemorrhagic stroke (ICD10: I60-I62)<br>Unspecified stroke (ICD10: I64-I69)	☐<br>☐<br>☐<br>☐
A9	What number of stroke patients are usually in your hospital at any one time?	Approximate number (cases)	☐

Page 1 of 6

A. Hospital Characteristics		
A10	Which of the following departments are usually involved in providing care for stroke patients during their acute illness? (Tick all that apply)	General (internal) medicine ☐<br>Neurology ☐<br>Surgery ☐<br>Rehabilitation ☐<br>General practice / Family medicine ☐<br>Nursing ☐<br>Pharmacy ☐<br>Other (specify) ________ ☐
A11	What proportion of stroke patients are looked after by a specialist doctor training in stroke?	approximate proportion (%) ☐
A12	Does your hospital have your own stroke registry?	☐ Yes ☐ No
A13	Does your hospital have any network with other hospitals and which hospital is the main hub?	☐ Yes, having stroke fast track Hospital hub name: ________ ☐ No<br>☐ Yes, having referral system Hospital hub name: ________ ☐ No

B. Healthcare service funding					
B1	What proportion (%) of source of fund for stroke services?	Services	Government funding	Private funding	Other (specify)
		Medical services			
		Medicine (i.e. rt-PA drug)			
		Investigation (i.e. CT scanning, MRI, lab test)			
		Rehabilitation programme			
		Material and equipment			
		Education programme			
		Other (specify) ________			
B2	Which items would the patient have to pay for?	☐ Medical services<br>☐ Medicines<br>☐ Medical equipment<br>☐ Other (specify) ________	☐ Investigations (e.g. CT scanning, MRI)<br>☐ Therapy (e.g. physiotherapy)<br>☐ None of the above/Free-of-charge		

C. Stroke unit services		
C1	Does your hospital have a Stroke Unit/corner?	Stroke Unit ☐ Yes (Go to C2) ☐ No (Go to C7)<br>Stroke corner ☐ Yes (Go to C2) ☐ No (Go to C7)
C2	How many beds are in your stroke unit/corner?	Number of beds in stroke unit ☐<br>Number of beds in stroke corner ☐
C3	What proportion (%) of stroke patients are usually admitted to stroke unit/corner?	approximate proportion (%) ☐

C. Stroke unit services		
C4	Which of these features describe your stroke unit?	☐ Discrete ward area for stroke patients<br>☐ Physicians and/or nurses whose work is mainly managing stroke patients<br>☐ Multidisciplinary team with main responsibility in managing stroke patients<br>☐ Special education programmes provided for stroke unit staff<br>☐ Information and education for stroke patients and their families or caregivers<br>☐ Written standard protocols for stroke management (i.e. a written management care plan)<br>☐ Other (specify) ___________
C5	Does your hospital receive a stroke centre certification?	☐ Yes ☐ No
C6	Which of the following staffs and number of staff are usually involved in providing care for stroke patients in stroke unit?	(see table below)

Type of staff	Approx. number of staffs in stroke unit	Number of part-time staffs	Number of full-time staffs
General practice			
Internal medicine			
Neurology			
Certificate in fellowship training in stroke			
Surgery			
Neurosurgery			
Emergency medicine			
Radiology/ Interventional radiology			
Rehabilitation physician			
Family medicine			
Nurse			
Pharmacist			
Physiotherapist			
Occupational therapist			
Speech-Language therapist			
Psychologist			
Social worker			
Other (specify) ___________			

C. Stroke unit services		
C7	Do you have a multidisciplinary team (medical, nursing, pharmacy, therapy staff) who meet on a regular basis to plan patient management?	☐ Yes, meeting once per day<br>☐ Yes, meeting once per week<br>☐ Yes, meeting less than once per week (specify) ______<br>☐ No multidisciplinary team
C8	After initial acute treatment, where do patients usually receive continuing rehabilitation in hospital?	☐ Yes, in general medical or neurology wards<br>☐ Yes, in the stroke unit<br>☐ Yes, transfer to another hospitals (higher level)<br>☐ Yes, transfer to another hospitals (lower level)<br>☐ No continuing rehabilitation available<br>☐ Other (specify) ______
C9	If patients require ongoing rehabilitation at the time of discharge from hospital, who usually delivers care to these patients and how are they funded?	(see table below)

Staff	Government funded	Privately funded	Other (specify)
Doctors			
Nurses			
Physiotherapists			
Occupational therapists			
Speech therapists			
Psychologists			
Village Health Volunteer			
Family members	- - - - - - - - -		
Other (specify) ______			

C10	Are the patient or family members / care givers usually educated in rehabilitation?	☐ Yes ☐ No (Go to D1)
C11	Who usually educates patient or family members / care givers in rehabilitation?	☐ Doctors ☐ Nurses<br>☐ Physiotherapists ☐ Occupational therapists<br>☐ Psychologists ☐ Speech therapists<br>☐ Village Health Volunteer ☐ Other (specify) ______

D. Other facilities and services		
D1	Do you have a written guidance for stroke management (i.e. a written management care plan) for hospital staffs?	☐ Yes ☐ No (Go to D4)
D2	Which of the topics are included in your guidance?	Please list any items/topics included in your hospital protocols *(e.g. assessment of swallowing impairment, early mobilisation, prevention of complications)*

Page 4 of 6

D. Other facilities and services

D3 Can you provide written copies of the guidance if requested?

☐ Yes ☐ No

D4 Which and when of these clinical assessment scores does your hospital uses for evaluating stroke patients? And where do you record these data (i.e. electronic database, OPD card)?

Admission	Discharge	Recorded location	Clinical assessment
			Glasgow coma scales
			Barthel Index
			modified Rankin Score
			National Institutes of Health Stroke Scale
			Swallowing impairment
			Level of consciousness
			Malnutrition
			Other (specify) ______________

D5 Which and when of these laboratory investigations does your hospital uses for investigating stroke patients? And where do you record these data (i.e. electronic database, OPD card)?

Admission	Discharge	Recorded location	Laboratory
			Blood sugar
			HbA1C
			CBC
			INR
			Creatinine
			EGFR
			Total cholesterol
			LDL
			HDL
			Triglyceride
			Other (specify) ______________

D6 Which of these medications are provided and what proportion (%) of stroke patients are usually received each drug?

Medications	Approximate proportion (%)
Recombinant tissue plasminogen activator (rt-PA)	
Antiplatelet (i.e. aspirin, ticlopidine, clopidogrel, aspirin + dipyridamole)	
Anticoagulant (i.e. warfarin, dabigatran, apixaban, rivaroxaban)	

D7 If you have a rt-PA drug, which route of rt-PA is prescribed?

☐ Intravenous ☐ Intra-arterial
☐ Other (specify) ______________________________

D8 Which investigations and what proportion (%) of stroke patients are usually received within 24 hours after admission in your hospital?

Investigations	Approximate proportion (%)
Elektrokardiogram (EKG) monitor	
Computerized Tomography (CT) brain	
Magnetic Resonance Imaging (MRI)/Angiography (MRA)	
Carotid Doppler ultrasound	
Other (specify) ______________________	

D. Other facilities and services		
D9	Does your hospital can perform an endovascular treatment (thrombectomy) in acute stroke?	☐ Yes ☐ No
D10	Do you have a prevention of complications written plans/protocols?	☐ Yes ☐ No

E. Post-stroke care			
E1	Does your hospital have a post-rehabilitation unit?	☐ Yes ☐ No	
E2	Which type of medical rehabilitation services are provided and what proportion of stroke patients are received this service?	**Rehabilitation services**	**approximate proportion (%)**
		Acute/Inpatient rehabilitation	
		Outpatient rehabilitation	
		Post-acute rehabilitation	
		Home-based rehabilitation	
		Community-based rehabilitation	
		Others (specify) ___	
E3	Do you have a follow-up system after patient discharge from hospital?	☐ Yes ☐ No	
E4	Which type of home health care services are provided for stroke patients?	☐ GP / Internal medicine<br>☐ Family medicine<br>☐ Rehabilitation physician<br>☐ Pharmacist<br>☐ Physiotherapist<br>☐ Speech-language pathologist<br>☐ Village Health Volunteer<br>☐ Thai Traditional Medicine	☐ Neurologist / Certificate in fellowship training in stroke<br>☐ Emergency medicine<br>☐ Nurse<br>☐ Psychologists<br>☐ Occupational therapist<br>☐ Nutritionist<br>☐ Social Medicine<br>☐ Others (specify) ___
E5	Does your hospital have an intermediate care service (IMC) and long-term care (LTC) service?	IMC<br>☐ Yes ☐ No<br>LTC<br>☐ Yes ☐ No	

Further comments

....

....

....

THANK YOU VERY MUCH

Please return the questionnaire via email: 2425416K@student.gla.ac.uk

Date of completing questionnaire ___/_____/____

Page **6** of **6**

Figure S2 Stroke questionnaire adapted from the INTERSTROKE study

3. Pilot test and revision

Before circulating the questionnaire, it was reviewed by two non-academic healthcare staff from hospitals (neurologists) and one academic staff member. After revision, a pilot questionnaire was sent to four secondary hospitals to help ensure their understanding and ability to complete the questions. Finally, the questionnaire sentences/wording was revised based on their comments.

4. Ethical consideration

The questionnaire and related documents such as inform consent, definition, research summary, ethical approval certificate, were presented in Thai language and this study was submitted and granted by the Ethical Review Committee of the Institute for Development of Human Research Protection, MOPH (No.IHRP722/2562) prior to conducting this study. All research was performed in accordance with the declaration of Helsinki ethical principles. Ethical approval was granted for public hospitals under the Thai MOPH (12 health regions).

5. Questionnaire distribution and administration

Prior questionnaire distribution, the author performed a telephone call to ask for key managerial health professionals - health professionals who work or have main responsibility related to stroke service provision in their hospital to be the respondents/coordinators. For example, nurse case manager, neurologist. Then, a brief introduction of this study was presented and asked if they could provide their contact information such as telephone number or email. Next, the self-completed questionnaire were distributed via e-mail (PDF-fillable form questionnaire), online-questionnaire (using paid web-based survey), paper questionnaire (by post). Telephone calls were offered if clarification was required by respondents. Online-questionnaires were created using SurveyGizmo® website (collaborator license). The full-set of online-questionnaire was available at https://sgiz.eu/s3/Stroke-TH.

When participants answered in paper questionnaire format, a free business-reply return envelope were included when sending a post to hospitals. In terms of online questionnaire, the questionnaire answers were recorded electronically. Data from the online-questionnaire were then exported into a Microsoft Excel file format with coded responses. The author combined both answers from paper and online questionnaires, then checking for consistency and made a telephone call back to the respondents if there were some errors or needed a confirmation or clarification. Data were entered or exported into Microsoft Excel spreadsheets before being transferred to R for analysis.

6. Data collection

Table S4 shows the timeline of data collection procedure, after distributing the self-reported questionnaire (day 0), researcher informed the respondents by e-mail or telephone within 7-14 days to confirm whether letter to hospital director, questionnaire and related documents were received and registered into their systems. A one-month follow-up asked whether participants had received all related documents. Then, researcher waited for 4 weeks

and confirm whether they had received and completed it, afterwards, e-mail was resent and telephone calls and social applications were made with the option to complete the questionnaire by using telephone interview to increase the response rate. Next, telephone calls and email were performed every two weeks for reminding and stimulating the respondents. Subsequently, the author started analysing data within month 5th and 6th months. Finally, this study was closed at month seven. Data were collected for the fiscal year 2018 (1 October 2017 - 30 September 2018). First author (SK) collected and managed for survey anonymity and confidentiality of these data. A 10% of records was randomly selected for double check by one co-author (CG).

Table S4 Data collection timeline

Period	Date	Detail
Day 0	1-Nov-2019	Distribution of the self-reported questionnaire to all hospitals by post and online questionnaire channels.
Day 7-14	15-Nov-2019	- Telephone call to a confirmation whether questionnaire was registered into the hospital systems and whether the participants received questionnaire.<br>- Distribution of the self-reported questionnaire by E-mail to coordinator/respondents who had already received a permission from their hospital directors.
Day 30	30-Nov-2019	Telephone call to a confirmation whether the participants received questionnaire and had completed it
Day 31-60	30-Dec-2019	- Data collection and data confirmation<br>- A follow-up email was sent and a telephone call for reminding and stimulating the respondents
Every 2 weeks		- Data collection and data confirmation<br>- A follow-up email was sent and a telephone call for reminding and stimulating the respondents
Month 5th	30-Mar-2020	- Data collection (extending due to Coronavirus-19 outbreak)
Month 6th	30-Apr-2020	- Data collection<br>- Data management and analysis
Month 7th	30-May-2020	- Study closed<br>- Data management and analysis

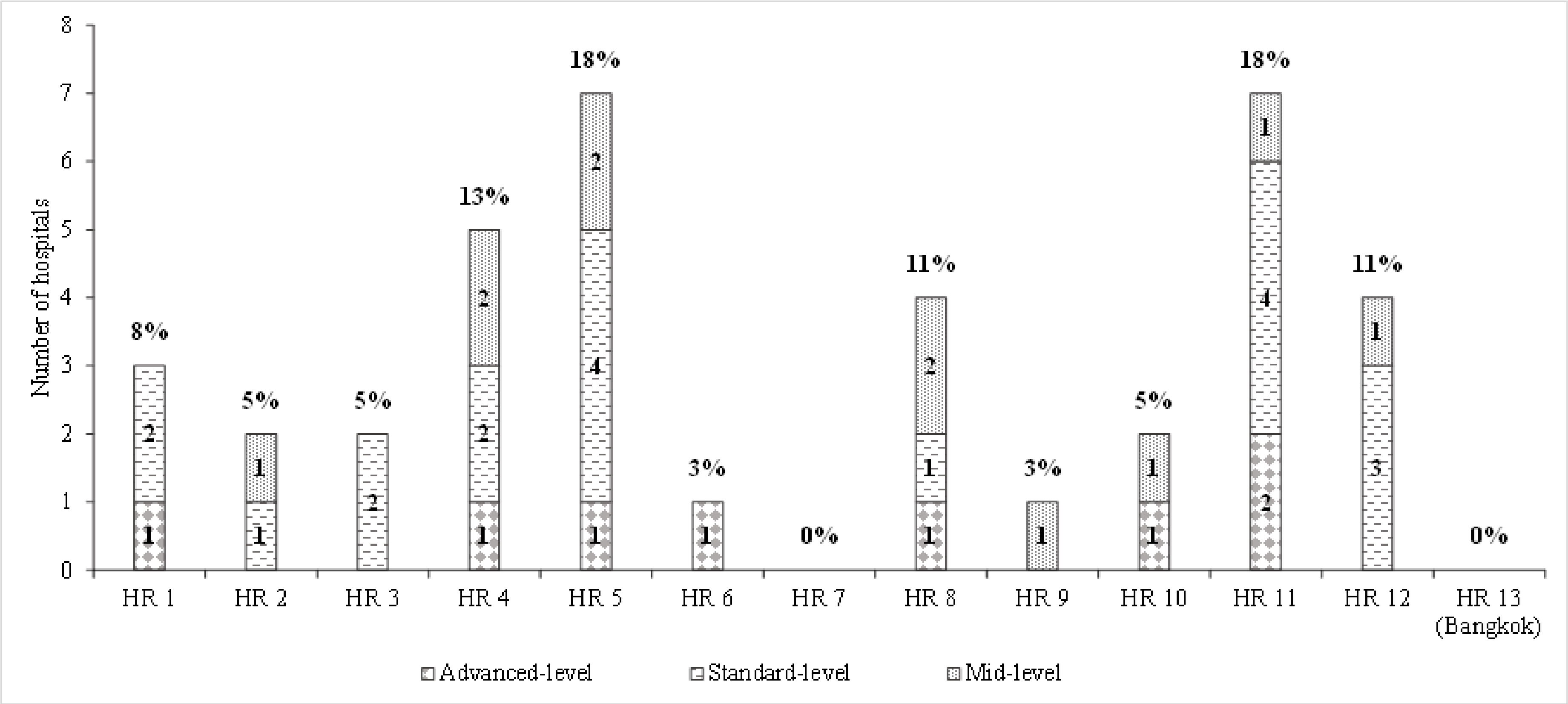

Figure S3 Number of questionnaires received and proportion of questionnaires (N=38)

Advanced-level: Advanced-level referral hospitals, HR: Health region, Mid: Mid-level referral hospitals (M1-level), Standard-level: Standard-level referral hospitals

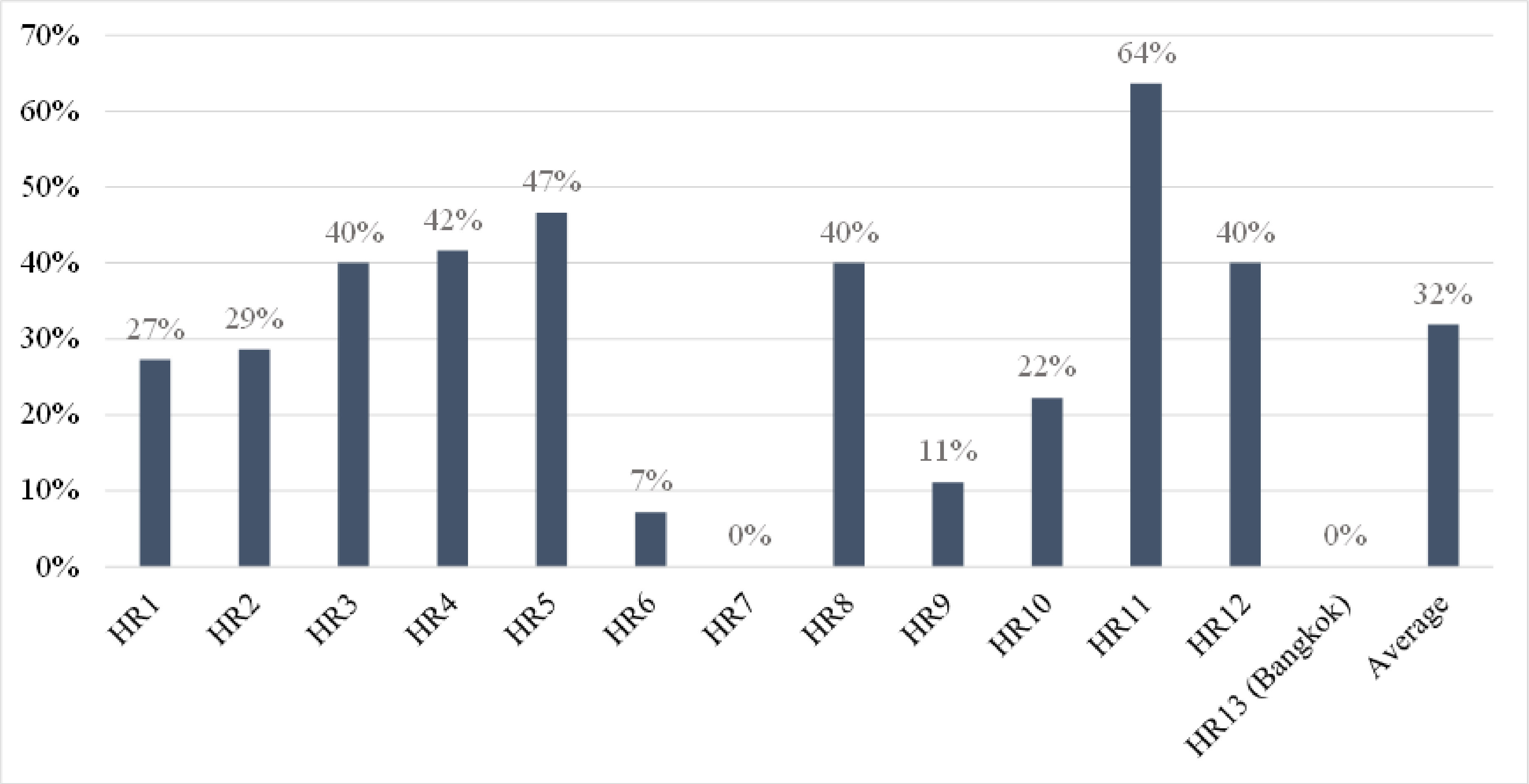

Figure S4 Response rate, by health region (N=38)

HR: health region

Table S5 Type and number of staff providing care for stroke patients in stroke unit/stroke corner

Type of staff		Advanced-level referral hospitals (N=6)			Standard-level referral hospitals (N=17)			Mid-level referral hospitals (N=10)		
		**All**	**FT**	**PT**	**All**	**FT**	**PT**	**All**	**FT**	**PT**
**Internal medicine**	**N**	**3**	**3**	**-**	**14**	**9**	**6**	**10**	**8**	**3**
	Median (IQR)	4 (5)	4 (5)	-	2 (4)	4 (5)	1 (-)	4 (3)	2 (3)	5 (1)
	min-max	1-12	1-12	-	1-7	1-7	1-2	1-7	1-5	4-6
**Neurology**	**N**	**6**	**5**	**1**	**5**	**3**	**2**	**-**	**-**	**-**
	Median (IQR)	2 (2)	1 (2)	2	1	1	1	-	-	-
	min-max	1-3	1-3	-	1	1	1	-	-	-
**Neurosurgery**	**N**	**6**	**2**	**4**	**8**	**3**	**5**	**1**	**-**	**1**
	Median (IQR)	3 (1)	2 (1)	3 (-)	1 (-)	2 (-)	1 (1)	1	-	1
	min-max	1-4	1-2	3-4	1-2	1-2	1-2	-	-	-
**Radiology**	**N**	**3**	**1**	**2**	**8**	**5**	**3**	**7**	**5**	**2**
	Median (IQR)	5 (4)	1	7 (1)	2 (1)	2 (1)	2 (1)	2 (1)	2 (1)	2
	min-max	1-8	-	5-8	1-3	1-2	1-3	1-2	1-2	-
**Rehabilitation physician**	**N**	**5**	**3**	**3**	**13**	**7**	**6**	**4**	**3**	**1**
	Median (IQR)	2 (2)	1 (2)	1 (1)	1 (1)	1 (1)	1 (1)	1 (1)	1 (1)	1
	min-max	1-5	1-5	1-2	1-2	1-1	1-1	1-1	1-1	-
**Nurse**	**N**	**6**	**5**	**2**	**16**	**14**	**3**	**9**	**9**	**1**
	Median (IQR)	13 (8)	10 (8)	13 (9)	8 (6)	8 (7)	8 (8)	8 (5)	8 (5)	2
	min-max	7-22	7-18	3-22	2-20	2-15	4-19	3-18	1-18	-
**Physiotherapist**	**N**	**6**	**5**	**1**	**14**	**7**	**8**	**10**	**8**	**3**
	Median (IQR)	1 (1)	1 (1)	2	1 (2)	1 (2)	2 (2)	4 (3)	3 (2)	5 (3)
	min-max	1-2	1-2	-	1-7	1-7	1-7	1-6	1-6	1-6

Type of staff		Advanced-level referral hospitals (N=6)			Standard-level referral hospitals (N=17)			Mid-level referral hospitals (N=10)		
		**All**	**FT**	**PT**	**All**	**FT**	**PT**	**All**	**FT**	**PT**
**Social worker**	**N**	**4**	**3**	**1**	**11**	**2**	**9**	**9**	**6**	**3**
	Median (IQR)	1	1	1	1	1	1	1	1	1
	min-max	1	1	-	1-2	1	1-2	1-2	1-2	1

FT: full time staff, N: Number of hospitals, PT: part time staff, IQR: inter quartile range
All: Approx. number of all staff; Full-time staff: staffs who provide care for stroke patients in stroke unit/stroke corner at least 40 hours per week; Part-time staff: staffs who provide care for stroke patients in stroke unit/stroke corner less than 40 hours per week.

Reference

1. Bureau of Health Administration. Service plan strategies 2018-2022. Nonthaburi: Ministry of Public Health; 2016.
2. JICA Research Institute, Saitama University and Thammasat University. Case Studies of Social Infrastructure Demand Estimates in Indonesia and Thailand.: Japan International Cooperation Agency (JICA) Research Institute; 2018. Available from: https://www.jica.go.jp/jica-ri/publication/booksandreports/20180831_02.html.
3. Jongudomsuk P, Srithamrongsawat S, Patcharanarumol W, Limwattananon S, Pannarunothai S, Vapatanavong P, et al. The Kingdom of Thailand Health System Review. World Health Organization, Regional Office for the Western Pacific. 2015.
4. Bureau of Health Administration. Operating the service plan policies, 2017-2021. Nonthaburi: Office of the Permanent Secretary, Ministry of Public Health; 2017.
5. Langhorne P, O'Donnell MJ, Chin SL, Zhang H, Xavier D, Avezum A, et al. Practice patterns and outcomes after stroke across countries at different economic levels (INTERSTROKE): an international observational study. The Lancet. 2018;391(10134):2019-27.
